# ECMO as a bridge to heart transplantation: Insights into stratification by heart failure etiology

**DOI:** 10.1016/j.jhlto.2024.100097

**Published:** 2024-04-26

**Authors:** Manuj M. Shah, Hannah Rando, Antonio R. Polanco, Ahmet Kilic

**Affiliations:** Division of Cardiac Surgery, Department of Surgery, Johns Hopkins Hospital, Baltimore, Maryland

**Keywords:** ECMO, heart transplantation, heart failure etiology, bridge to transplant, post-transplant outcomes

## Abstract

**Background:**

Revisions to the heart allocation criteria in 2018 motivated an increased use of extracorporeal membrane oxygenation (ECMO) as a bridge to transplantation. Studies have demonstrated inferior post-transplant outcomes in patients bridged with ECMO but do not account for underlying diagnosis. Our objective was to elucidate the differential impact of ECMO on outcomes by heart failure (HF) etiology.

**Methods:**

The United Network of Organ Sharing database was queried for adults who underwent isolated heart transplantation after October 2018. Patients were stratified by ECMO utilization at the time of transplantation and then by HF etiology. After baseline statistical comparisons, survival analysis relied on Kaplan-Meier estimates and Cox proportional models.

**Results:**

A total of 13,203 patients were included, of whom 761 (5.8%) were supported with ECMO. ECMO patients were younger (48 vs 54 years, *p* < 0.001), less likely to have diabetes (24% vs 30%, *p* < 0.001), smoke cigarettes (31% vs 41%, *p* < 0.001), or have prior cardiac surgery (29% vs 36%, *p* < 0.001), more likely to require dialysis (20% vs 5%, *p* < 0.001), and spent fewer days on the waitlist (59 vs 190, *p* < 0.001). After adjustment, ECMO was associated with increased mortality (hazard ratio 1.85, *p* < 0.001) in the full cohort. After incorporating HF etiology, this increased mortality risk persisted in all subgroups except restrictive cardiomyopathy and congenital heart disease (CHD).

**Conclusions:**

Our findings illustrate that HF etiology is associated with differing outcomes when bridging with ECMO. ECMO patients with restrictive cardiomyopathy or CHD did not have increased mortality risk. With ECMO utilization increasing, these data are hypothesis-generating and serve as a basis for further studies.

## Background

Heart disease is the leading cause of death worldwide, contributing to significant morbidity and mortality in our growing, aging population.^1^, ^2^ Heart transplantation is widely recognized as the gold standard and only curative option for patients with advanced heart failure, particularly when patients are unresponsive to conventional medical therapies and interventions.^1^, ^3^, ^4^ However, with an increasing prevalence of heart disease and shortage of donor hearts, the number of candidates awaiting transplantation continues to grow. This has necessitated the development and utilization of advanced therapies, such as durable mechanical circulatory support (MCS) to sustain waitlist candidacy until a donor heart is available. Another strategy is the utilization of temporary MCS as a bridge to transplantation and if biventricular support is needed and/or patients are in extremis, to utilize veno-arterial extracorporeal membrane oxygenation (ECMO).

ECMO functions by circulating deoxygenated blood out of the venous system through an oxygenator and pump to generate perfusion back to the arterial side of the body. Similar to other temporary MCS devices, ECMO aims to support cardiopulmonary function and improve end-organ dysfunction to promote organ recovery and maintain waitlist eligibility in transplant candidates. Although the use of all temporary MCS modalities has increased over the past decade, ECMO use as a bridge to heart transplantation has skyrocketed from 1.7% in 2010 to 6.1% in recent years.^5^, ^6^, ^7^ With the revised allocation criteria from the United Network for Organ Sharing (UNOS), patients on ECMO retain the highest priority on the waitlist, perhaps motivating the progressive increase in ECMO use.^8^ The criteria modifications took effect on October 18, 2018, in hopes of decreasing waitlist mortality, improving equity of donor hearts, and prioritizing critically ill patients on the waitlist for transplant.^8^

Unfortunately, ECMO is associated with greater morbidity and mortality relative to other MCS strategies and has a high frequency of complications, such as major bleeding and embolic stroke.^9^, ^10^, ^11^ Though rates of clinical deterioration on the waitlist and post-transplant mortality in patients on ECMO have improved, there remains uncertainty about which patients benefit most from ECMO.^12^ Nevertheless, it remains a vital tool to support hemodynamic function before, during, and after surgical intervention.^3^, ^13^ As ECMO use continues to increase and the heart transplant population becomes older and sicker, we recognize a need to investigate and understand what specific patient profile derives the most meaningful benefit from ECMO and delineate potential reasons behind these findings.

Given the wide range of indications for heart transplant and the heterogeneity in patient physiology, we reason that the underlying disease profile may influence patient outcomes. Previous research has demonstrated that regardless of ECMO status, patients with restrictive cardiomyopathy (CMP), congenital heart disease (CHD), and prior heart transplantation were less likely to survive transplant compared to other diagnoses.^14^ Additional literature has characterized other risk factors, such as older age and history of prior cardiac surgery, for adverse post-transplant outcomes in patients bridged with ECMO.^15^, ^16^, ^17^, ^18^ However, to our knowledge, there has not been any investigation into the impact of heart failure etiology on post-transplant morbidity and mortality with ECMO. In the present paper, we aim to characterize post-transplantation outcomes in patients bridged to transplantation on ECMO with the specific hypothesis that restrictive CMP, CHD, and retransplant patients bridged with ECMO would have inferior post-transplant outcomes.

## Materials and methods

### Study design and definitions

We performed a retrospective review using the UNOS database, which is a prospectively collected registry of all organ transplant candidates and recipients in the United States. The database was queried for all adult heart transplant recipients age ≥18 years old who underwent transplantation after the allocation change in October 2018. Recipients were excluded if they received multiorgan transplantation or if they had no follow-up data. This study was approved by the Johns Hopkins University Institutional Review Board (IRB00159748) and was in compliance with the International Society for Heart and Lung Transplantation ethics statement.

**Figure 1:**
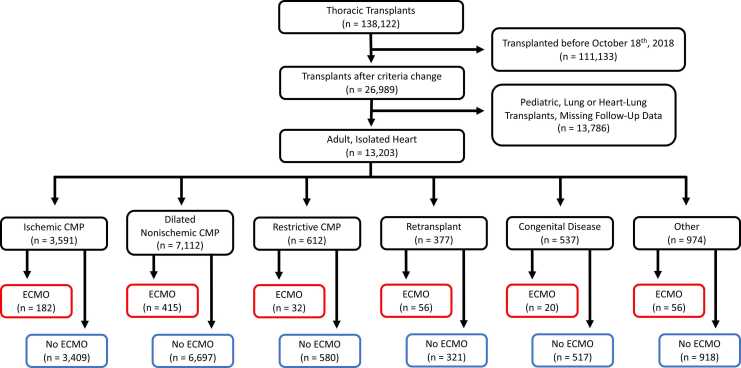
Diagram depicting the breakdown of inclusion and exclusion criteria to generate study population from the UNOS dataset. CMP, cardiomyopathy; ECMO, extracorporeal membrane oxygenation.

Patients were first stratified into 2 study arms based on their ECMO status at the time of transplantation: ECMO or no ECMO. Subsequent stratification was performed to categorize patients into 6 additional subgroups based on heart failure etiology: (1) ischemic CMP (including coronary artery disease), (2) dilated nonischemic CMP, (3) restrictive CMP, (4) retransplantation, (5) CHD, and (6) other, which includes hypertrophic CMP and valvular heart disease (Figure 1). Baseline characteristics were collected for all patients, including age, body mass index (BMI), sex, race/ethnicity, blood type, history of diabetes, cerebrovascular disease, cigarette use, prior cardiac surgery, cardiac support, preoperative dialysis, allocation status, days spent on the waitlist, and donor characteristics, namely age, sex, ischemic time, and predicted heart mass (PHM). In the UNOS database, race and ethnicity are reported such that any patient with Hispanic ethnicity is included in the Hispanic/Latino category. Patients were defined as having cardiac support if an intra-aortic balloon pump or any type of ventricular assist device was placed preoperatively.

Most recent clinical hemodynamic and laboratory data at the time of transplant were obtained for all patients, including serum creatinine, total bilirubin, mean pulmonary artery pressure (mPAP), systolic pulmonary artery pressure, diastolic pulmonary artery pressure (dPAP), pulmonary capillary wedge pressure (PCWP), and cardiac output. These values were only collected for patients with a completely intact record (i.e., patient was not missing any 1 of the 7 collected variables). Estimated glomerular filtration rate (eGFR) values were calculated using the collected serum creatinine according to the Chronic Kidney Disease Epidemiology Collaboration equation.^19^ A sensitivity analysis was performed to compare baseline characteristics of patients missing any laboratory data. Lastly, for up to 1-year post-transplantation, follow-up data regarding patient mortality and complications after transplantation were collected, including acute rejection, dialysis status, graft status, hospitalization for infection, and pacemaker insertion. Graft failure was documented for any removal of the transplanted heart, recipient death, or placement on a chronic allograft support system.

### Statistical analysis

Baseline characteristics, hemodynamic data, laboratory values, and post-transplant outcomes were compared between ECMO patients and no ECMO patients using Student's *t*-tests or Wilcoxon rank-sum tests for continuous variables and chi-square tests or Fisher's exact tests for categorical variables, as appropriate. Analogous comparisons were then made between ECMO patients and no ECMO patients within each diagnosis subgroup.

Survival analysis was performed using Kaplan-Meier estimates for up to 1-year post-transplantation; log-rank tests were used to compare ECMO patients vs no ECMO patients, first in the whole study cohort and then within each subgroup. Cox proportional hazard analyses were then carried out to elucidate the relationship between ECMO and post-transplantation mortality after adjusting for potential confounders. Covariates were selected a priori based on clinical knowledge and a literature review of risk factors for post-transplant mortality; these were age, BMI, months on waitlist, preoperative dialysis, diabetes, prior cardiac surgery, cigarette use, donor age, donor sex, ischemic time, and PHM. Stratified log-log survival curves were used to confirm that the proportional hazards assumption was not violated (Figure S1).

All statistical analysis was carried out using STATA/IC 16.1 (StataCorp LLC, College Station, TX). Significance was set to a *p*-value ≤0.05 for all tests.

## Results

### Patient demographics and baseline characteristics

A total of 13,203 patients were identified to meet the inclusion criteria, of whom 761 (5.8%) were supported with ECMO at the time of transplantation (Figure 1). Overall, patients on ECMO were younger (mean 47.7 vs 53.6, *p* < 0.001), had a lower BMI (mean 27.36 vs 27.74, *p* = 0.047), and were more likely to be male (76.6% vs 72.9%, *p* = 0.026) (Table 1). ECMO patients were also less likely to have diabetes (23.8% vs 30.0%, *p* < 0.001) or smoke cigarettes (31.3% vs 41.0%, *p* < 0.001), less likely to have a prior cardiac surgery (28.5% vs 36.3%, *p* < 0.001) or cardiac support (54.5% vs 64.0%, *p* < 0.001), more likely to require preoperative dialysis (19.8% vs 5.4%, *p* < 0.001), and spent fewer days on the waitlist (mean 59.2 vs 190.2, *p* < 0.001) (Table 1). Additionally, the donors for ECMO patients were younger (mean 31.1 vs 32.5, *p* < 0.001), more likely to be male (82.7% vs 71.8%, *p* < 0.001), had shorter ischemic times (mean 3.4 vs 3.5, *p* = 0.039), and greater PHM (mean 193.4 vs 186.2, *p* < 0.001) (Table 1). The most common heart failure etiologies were dilated nonischemic and ischemic CMP in both groups, but ECMO patients were slightly less likely to have ischemic CMP and were more likely to be undergoing retransplantation (Table 1).

**Table 1:** Demographics and Baseline Characteristics for All Patients by ECMO Status at the Time of Transplantation

Variable	ECMO	No ECMO	*p*-value
	N = 761	N = 12,442	
Age	47.7 (14.5)	53.6 (12.9)	<0.001
BMI	27.36 (5.24)	27.74 (5.02)	0.047
Sex			
Female	178 (23.4%)	3,370 (27.1%)	0.026
Male	583 (76.6%)	9,072 (72.9%)	
Race/ethnicity			
White	438 (57.6%)	7,466 (60.0%)	<0.001
Black	191 (25.1%)	3,098 (24.9%)	
Hispanic	64 (8.4%)	1,278 (10.3%)	
Asian	53 (7.0%)	456 (3.7%)	
Other	15 (2.0%)	144 (1.2%)	
Blood type			
A	276 (36.3%)	4,885 (39.3%)	0.33
B	42 (5.5%)	625 (5.0%)	
AB	115 (15.1%)	1,919 (15.4%)	
O	328 (43.1%)	5,013 (40.3%)	
Diabetes	181 (23.8%)	3,726 (30.0%)	<0.001
Cerebrovascular disease	43 (5.7%)	938 (7.6%)	0.055
Cigarette use	238 (31.3%)	5,102 (41.0%)	<0.001
Prior cardiac surgery	217 (28.5%)	4,511 (36.3%)	<0.001
Cardiac support	415 (54.5%)	7,968 (64.0%)	<0.001
Preoperative dialysis	151 (19.8%)	674 (5.4%)	<0.001
Allocation score			
1	697 (91.6%)	563 (4.5%)	<0.001
2	56 (7.4%)	6,384 (51.3%)	
3	4 (0.5%)	2,269 (18.2%)	
4	4 (0.5%)	2,505 (20.1%)	
5	0 (0.0%)	115 (0.9%)	
6	0 (0.0%)	606 (4.9%)	
Days on waitlist	59.2 (289.4)	190.2 (391.7)	<0.001
Heart failure group			
Ischemic	182 (23.9%)	3,409 (27.4%)	<0.001
Dilated nonischemic	415 (54.5%)	6,697 (53.8%)	
Restrictive	32 (4.2%)	580 (4.7%)	
Retransplant	56 (7.4%)	321 (2.6%)	
Congenital	20 (2.6%)	517 (4.2%)	
Other	56 (7.4%)	918 (7.4%)	
Donor age	31.1 (9.4)	32.5 (10.3)	<0.001
Donor sex			
Female	132 (17.3%)	3,513 (28.2%)	<0.001
Male	629 (82.7%)	8,929 (71.8%)	
Donor ischemic time	3.4 (0.9)	3.5 (1.2)	0.039
Donor PHM	193.4 (30.0)	186.2 (33.1)	<0.001

Abbreviations: ECMO, extracorporeal membrane oxygenation; PHM, predicted heart mass.

Standard deviation for continuous variables and percentage of sample for categorical variables are displayed in parentheses, as appropriate. Donor ischemic time is presented in hours.

When performing the same comparisons within each heart failure group, many of these differences persisted, with several notable exceptions (Table S1). Among patients with restrictive CMP, ECMO patients had a higher BMI than no ECMO patients (mean 28.9 vs 26.8, *p* = 0.022). Furthermore, within the retransplant cohort, ECMO patients were older than no ECMO patients (mean 48.1 vs 43.4 years, *p* = 0.037), more likely to have smoked cigarettes (33.9% vs 19.9%, *p* = 0.020), and more likely to have had cardiac support (48.2% vs 32.1%, *p* = 0.019) (Table S1).

### Patient hemodynamic and laboratory data

Within our study cohort of 13,203 patients, 544 ECMO patients (71.5%) and 11,174 no ECMO patients (89.8%) were identified to have complete hemodynamic and laboratory data, which are listed in Table 2. Comparison of baseline characteristics within ECMO patients with missing data compared to those without was largely unremarkable (Table S2). Overall, patients on ECMO had a higher eGFR at transplant (mean 79.66 vs 68.55, *p* < 0.001), higher total bilirubin (mean 1.84 vs 0.95, *p* < 0.001), higher mPAP (mean 29.10 vs 27.35, *p* < 0.001), higher dPAP (mean 21.68 vs 19.50, *p* < 0.001), higher PCWP (mean 21.04 vs 18.13, *p* < 0.001), and lower cardiac output (mean 4.35 vs 4.44, *p* < 0.001) (Table 2). Upon isolating patients who did not receive preoperative dialysis, eGFR at transplant remained higher in ECMO patients vs no ECMO patients (mean 84.4 vs 70.3, *p* < 0.001) (Table 2). These trends mostly persisted when stratifying patients into the heart failure subgroups (Table S3). Notably, the trend of higher mPAP, dPAP, and PCWP in ECMO patients was significant only within the dilated nonischemic CMP cohort, likely due to being the largest subpopulation (Table S3).

**Table 2:** Laboratory and Hemodynamic Data for All Patients by ECMO Status at the Time of Transplantation

Variable	ECMO	No ECMO	*p-value*
	N = 544	N = 11,174	
eGFR at transplant (ml/min/m^2^), all patients	79.66 (34.77)	68.55 (27.80)	<0.001
eGFR at transplant (ml/min/m^2^), patients without dialysis	84.38 (34.13)	70.28 (26.46)	<0.001
Total bilirubin at transplant (mg/dl)	1.84 (2.76)	0.95 (1.63)	<0.001
Mean pulmonary artery pressure (mm Hg)	29.10 (10.39)	27.35 (10.27)	<0.001
Systolic pulmonary artery pressure (mm Hg)	40.82 (14.16)	40.08 (14.17)	0.116
Diastolic pulmonary artery pressure (mm Hg)	21.68 (9.25)	19.50 (8.80)	<0.001
Pulmonary capillary wedge pressure (mm Hg)	21.04 (9.45)	18.13 (8.83)	<0.001
Cardiac output (liter/min)	4.35 (1.89)	4.44 (1.43)	<0.001

Abbreviations: ECMO, extracorporeal membrane oxygenation; eGFR, estimated glomerular filtration rate.

eGFR was calculated using serum creatinine at the time of transplant according to the Chronic Kidney Disease Epidemiology Collaboration equation; eGFR is presented for all patients including those with preoperative dialysis and then for all patients without preoperative dialysis (i.e., dialysis omitted). Data presented include only patients with intact hemodynamic data (i.e., not missing any 1 of 7 variables collected). Standard deviation is displayed in parentheses.

### Follow-up analysis

Patient data regarding post-transplantation outcomes and complications, such as graft dysfunction, were first compared based on ECMO status and then characterized with multivariate regression (Table 3, Table S4). Among all patients, the most frequent complication was hospitalization for infection, which occurred in 18% of patients. ECMO patients were more likely to experience renal dysfunction requiring chronic dialysis before adjustment (5.3% vs 3.8%, *p* = 0.039) (Table 3, Table S4). There were otherwise no notable differences in post-transplant complications between ECMO and no ECMO patients. When evaluating the incidence of complications within each diagnosis group, there was a higher incidence of acute rejection requiring treatment in ECMO patients with ischemic CMP (8.8% vs 5.8%, *p* = 0.042) and a lower incidence in ECMO patients undergoing retransplant (23.2% vs 36.1%, *p* = 0.029) relative to their no ECMO counterparts (Table S5). Within the CHD diagnosis subgroup, patients on ECMO were more likely to be hospitalized due to infection in the post-transplant period (40.0% vs 15.3%, *p* = 0.008) (Table S5).

**Table 3:** One-Year Post-Transplantation Outcomes and Complications for All Patients by ECMO Status at the Time of Transplantation

Variable	ECMO	No ECMO	*p*-value
	N = 761	N = 12,422	
Acute rejection			0.569
Yes, with antirejection treatment	62 (8.2%)	972 (7.8%)	
Yes, without treatment	48 (6.3%)	909 (7.3%)	
Renal dysfunction requiring dialysis	40 (5.3%)	469 (3.8%)	0.039
Graft dysfunction	33 (4.3%)	394 (3.2%)	0.077
Hospitalized for infection	133 (17.5%)	2,239 (18.0%)	0.718
Pacemaker placement	15 (2.0%)	292 (2.4%)	0.504

Abbreviation: ECMO, extracorporeal membrane oxygenation.

Percentage of sample is displayed in parentheses.

### Survival analysis

**Figure 2:**
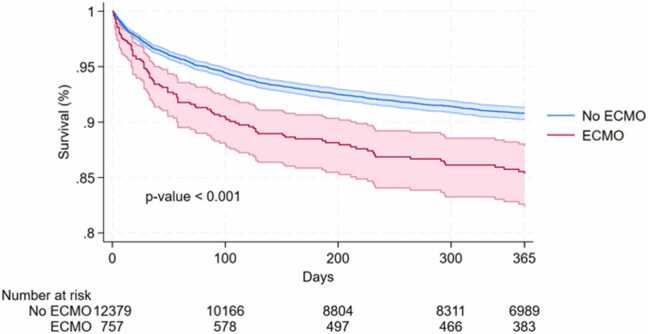
Kaplan-Meier survival estimates for all patients by ECMO status at the time of transplantation up to 1-year post-transplantation. The shaded area with borders for each curve shows 95% confidence interval. Survival axis is scaled from 0.80 to 1.00. *p*-value is shown in bottom left region of graph and was calculated using the log-rank test. ECMO, extracorporeal membrane oxygenation.

**Figure 3:**
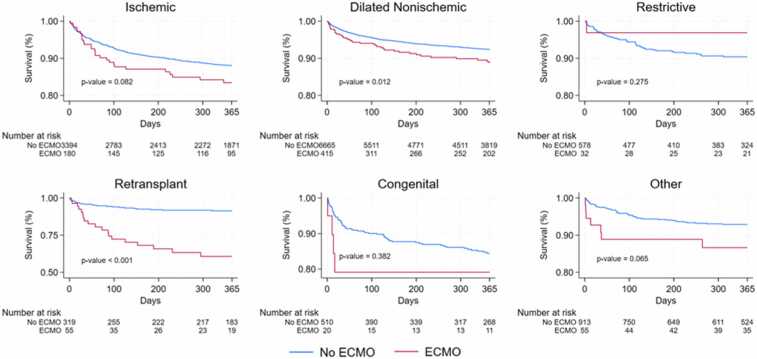
Kaplan-Meier survival estimates for all patients by ECMO status and heart failure etiology for up to 1-year post-transplantation. Survival axis is scaled from 0.80 to 1.00 (scaled from 0.50-1.00 for retransplant). Ninety-five percent confidence intervals are not shown. *p*-values are shown in the bottom left region of each graph and were calculated using the log-rank test. ECMO, extracorporeal membrane oxygenation.

Using Kaplan-Meier estimates and Cox proportional hazards regression, survival trends were analyzed for up to 1-year post-transplantation (Figures 2 and 3). In our full study cohort, ECMO at the time of transplantation was associated with a higher hazard of death in univariate analysis (hazard ratio [HR] 1.654, *p* < 0.001) (Table 4). However, after stratifying patients based on underlying heart failure etiology, ECMO was only associated with an increased hazard of death in 2 diagnosis subgroups: dilated nonischemic CMP (HR 1.518, *p* = 0.012) and retransplantation (HR 5.040, *p* < 0.001) (Table 4). Upon substratifying retransplantation patients into emergent and nonemergent cases using a threshold of 90 days between transplants, ECMO was associated with increased mortality even in the nonemergent cases of retransplantation.

**Table 4:** Cox Proportional Hazards Model Analysis for Risk of Mortality With ECMO for All Patients and by Heart Failure Etiology Before and After Multivariate Adjustment

Variable	Unadjusted			Adjusted		
	HR for ECMO	95% CI	*p*-value	HR for ECMO	95% CI	*p*-value
All patients	1.654	(1.343, 2.038)	<0.001	1.846	(1.488, 2.290)	<0.001
Ischemic	1.412	(0.955, 2.088)	0.084	1.591	(1.067, 2.371)	0.023
Dilated nonischemic	1.518	(1.094, 2.106)	0.012	1.907	(1.360, 2.675)	<0.001
Restrictive	0.349	(0.048, 2.525)	0.297	0.428	(0.056, 3.254)	0.412
Retransplant	5.040	(2.773, 9.163)	<0.001	3.435	(1.678, 7.030)	0.001
Congenital	1.561	(0.570, 4.277)	0.386	1.177	(0.406, 3.415)	0.764
Other	2.060	(0.940, 4.518)	0.071	2.947	(1.249, 6.958)	0.014

Abbreviations: BMI, body mass index; CI, confidence interval; ECMO, extracorporeal membrane oxygenation; HR, hazard ratio.

Covariates for multivariate adjustment include age, BMI, months on waitlist, preoperative dialysis, diabetes, prior cardiac surgery, cigarette use, donor age, donor gender, ischemic time, and donor-predicted heart mass.

After multivariate adjustment, the association between ECMO and mortality persisted. In the full study cohort, ECMO was associated with nearly a 2 times greater hazard of death (HR 1.846, *p* < 0.001) (Table 4). Additionally, patients with ischemic CMP had increased risk of mortality with ECMO (HR 1.591, *p* = 0.023) (Table 4). ECMO was not associated with an increased hazard of death in only 2 categories, restrictive CMP (HR 0.428, *p* = 0.412) and CHD (HR 1.177, *p* = 0.764) (Table 4).

## Discussion

This study aimed to characterize the relationship between ECMO use and post-transplantation outcomes after accounting for underlying heart failure etiology. We found that using ECMO as a bridge to heart transplantation was generally associated with increased mortality, but that this relationship was not present for patients with restrictive CMP or CHD.

The relationship we observed between ECMO and increased post-transplant mortality agrees with much of the literature. While selected studies have demonstrated similar post-transplant survival for patients bridged on ECMO relative to the no ECMO population,^20^ the majority have shown that preoperative and perioperative ECMO is associated with higher morbidity and mortality.^9^, ^11^, ^21^, ^22^, ^23^ Though recent advancements in mechanical devices and sheath insertion techniques have decreased mortality with ECMO usage, there continues to be an associated inferior post-transplant survival, especially in the short-term following heart transplantation and even when compared to other forms of MCS.^7^, ^23^, ^24^ In our study cohort, patients on ECMO were younger, had a lower BMI, were less likely to have diabetes, and were less likely to have prior cardiac surgery or cardiac support. This agrees with previous research and clinical decision-making as ECMO is often considered for younger patients with fewer comorbidities in hopes of eliciting improved tolerance.^4^, ^11^ We also note that patients on ECMO spent fewer days on the waitlist, likely a product of the revised allocation criteria aiming to prioritize the most critically ill patients.^25^ Despite these favorable circumstances, patients on ECMO were nearly twice as likely to experience post-transplant mortality after multivariate adjustment.

One potential explanation for this discrepancy in post-transplant mortality is the impact of ECMO on patient physiology. In this study, ECMO patients were found to have a greater need for preoperative and postoperative dialysis, possibly due to reduced renal perfusion and lack of pulsatile blood flow on ECMO.^26^ Patients on ECMO were also noted to have increased total bilirubin after cannulation, which may reflect hemolysis from the ECMO circuit or indicate venous congestion and liver dysfunction.^27^, ^28^ Elevated mPAP, dPAP, and PCWP values were also observed, indicating a degree of pulmonary hypertension and decreased systolic function that could impair perfusion and cardiovascular function post-transplant.^29^, ^30^ These physiologic alterations, particularly coupled with the high incidence of major bleeding and stroke observed in ECMO patients, likely contribute to the observed trend of increased mortality.^10^, ^21^, ^22^, ^31^, ^32^

Upon stratification of our study cohort, we found differential post-transplantation outcomes associated with underlying heart failure etiology. This is consistent with literature outside of ECMO, which has reported variability in post-transplant survival based on diagnosis.^33^ In our study specifically, both restrictive CMP and CHD patients on ECMO appeared to not have increased mortality compared to those not on ECMO. Restrictive CMP is characterized by abnormal ventricular filling and increased myocardial stiffness with subsequent myocardium distortion and venous congestion yet normal or near-normal systolic function.^34^ Given the underlying mechanism of ECMO and increase in systemic afterload with elevated intrapulmonary pressures, we may reason that restrictive CMP patients potentially have superior tolerance of preoperative ECMO due to their preserved systolic function as opposed to other forms of heart failure, such as dilated cardiomyopathies. Within our study population, we also note that restrictive CMP patients on ECMO had lower usage of other cardiac support measures, perhaps indicative of poor candidacy for other forms of temporary or durable MCS and sooner initiation of ECMO before potential clinical deterioration, as well as lower rates of prior cardiac surgery, a risk factor for post-transplant mortality.^7^ Finally, we note that even nonbridged restrictive CMP patients tend to have lower post-transplant survival relative to other diagnoses.^35^, ^36^, ^37^, ^38^ Explanatory factors for this trend include restrictive CMP patients’ increased frailty, renal dysfunction, elevated pulmonary pressures, and inotrope use.^35^, ^36^, ^37^, ^38^ As such, the noninferiority of post-transplant mortality in ECMO-bridged patients may be even more noteworthy.

Within the CHD subgroup, there also appeared to be no increased risk of post-transplant mortality for patients on ECMO relative to nonbridged patients. We acknowledge that our analyses may be underpowered given this subgroup’s sample size, but until more robust data become available, our findings remain suggesting that ECMO is not associated with an increased mortality risk within CHD patients. Possible rationale for this observed trend includes that compared to the entire study cohort, these patients tended to be younger with fewer comorbidities and had lower usage of cardiac support, both of which have correlated with more favorable post-transplant outcomes.^39^, ^40^, ^41^ CHD patients on ECMO also spent fewer days on the waitlist compared to other CMP patients, suggesting that CHD patients may experience quicker ECMO initiation and shorter durations of ECMO.^42^ Within the CHD diagnosis subgroup, ECMO patients spent approximately 236 fewer days awaiting transplantation than their non-ECMO counterparts, which is the largest relative difference within any of the diagnosis subgroups. With this, ECMO appears to facilitate access to transplantation for adult CHD patients, who can otherwise experience longer waitlist times.^43^ Although no other studies have described a differential benefit for CHD patients on ECMO relative to non-CHD patients, favorable outcomes have been described in adult CHD patients with MCS as a whole.^44^, ^45^, ^46^ Reported factors that may account for adult CHD patients’ tolerance of ECMO include their younger age, lower prevalence of hypertension and renal dysfunction, more robust immune response, chronic adaptation to hypoxic and/or low flow states, and the comprehensive care that comes with a uniquely multidisciplinary team.^46^, ^47^

### Limitations

This study is retrospective by design, which comes with inherent limitations such as potential confounding, selection bias, and inability to determine causation. While the UNOS database contains many variables for many patients, there is limited granularity regarding certain parameters, such as illness severity and reason for ECMO initiation; we acknowledge that ECMO may be initiated for many reasons, including a need for more circulatory support, control of arrhythmias, or active cardiopulmonary resuscitation. Additionally, we were not able to adjust for covariates outside of the database and intangible factors, such as medical advancement and changes in clinical care, that may influence outcomes. Furthermore, in this study, patients on ECMO were defined by their ECMO status at the time of transplantation; thus, patients who were on ECMO during the waitlist period but not at the time of transplantation were not included in the ECMO cohort.

We stratified patients according to their ECMO status and heart failure etiology. This study does not take into account the heterogeneity within each diagnosis group, which may be high in certain subgroups such as CHD. Additionally, meaningful findings cannot be drawn from the “other” diagnosis subgroup, which is heterogeneous by nature; we were not able to further separate out these patients due to the limited sample size of those diagnoses. Moreover, while the ischemic and dilated nonischemic CMP subgroups were large, we observed smaller sample sizes in other subgroups that may have hindered finding differences when analyzing outcomes. Lastly, we recognize that this study focuses on post-transplant outcomes and does not consider survival to transplantation. Successful bridging to transplantation is a very important topic of interest that encompasses aspects, such as waitlist mortality or clinical deterioration, MCS escalation and duration, and complications suffered while on MCS, but remains outside the scope of this work; further investigation is warranted before clinical decisions are to be drawn from this present study. We hope to overcome some of the constraints with future analyses as more patient data becomes available.

## Conclusions

We present a novel study investigating the impact of ECMO and heart failure etiology on patient outcomes after heart transplantation. Given the persistence of donor organ shortage and an increasing waitlist population, it is increasingly important to understand and optimize the perioperative management of heart transplant candidates. We describe an association between ECMO use and increased post-transplant mortality but note that this relationship is not present in patients with restrictive CMP and CHD. Bridging with ECMO is a multidimensional process, and these findings suggest that other factors, such as heart failure etiology, may impact outcomes. As the use of ECMO in this indication becomes more mainstay, it is increasingly meaningful to understand the most important factors surrounding ECMO utilization and how ECMO can be employed successfully as a bridge to transplant. This present study serves as a basis for future investigation on this topic and a deeper understanding of this technology.

## CRediT authorship contribution statement

Shah, Rando, Polanco, and Kilic contributed to the design and implementation of the research and its main conceptual ideas. Shah processed the study population data, performed the analysis, drafted the manuscript, and designed the figures and tables. Rando verified the data analysis and helped with the interpretation of the findings and writing of the manuscript. Polanco assisted with critical review and the drafting of the manuscript. Kilic aided in factual review, commented on the manuscript, and overall helped to supervise the project.

## Disclosure statement

The authors declare that they have no known competing financial interests or personal relationships that could have appeared to influence the work reported in this paper.

We would like to express our sincere appreciation to all the individuals who have contributed to the completion of this research paper. We would like to thank the United Network for Organ Sharing for allowing us to work with their extensive patient database as our primary source of data. We are also grateful to Dr Sari Holmes with the Johns Hopkins Department of Cardiac Surgery who assisted and verified the statistical methods and analysis. We lastly would like to thank the International Society for Heart and Lung Transplantation for the opportunity to present our findings at the annual conference in April 2023.

There was no funding obtained for this study.
