# Supplementary material for: ECMO as a bridge to heart transplantation: Insights into stratification by heart failure etiology
Source: JHLT Open. 2024 Apr 26;5:100097. doi: 10.1016/j.jhlto.2024.100097 (PMC11935478; doi:10.1016/j.jhlto.2024.100097)
Supplement: Supplementary file 1 — Supplementary material [file mmc1.docx]

**Supplemental Legend:**

**Supplemental Table 1**: Demographics and baseline characteristics for all patients by ECMO status and heart failure etiology.

**Supplemental Table 2:** Comparison of baseline characteristics within ECMO patients based on whether hemodynamic and laboratory data was intact.

**Supplemental Table 3**: Laboratory and hemodynamic data at the time of transplantation for all patients by ECMO status and heart failure etiology.

**Supplemental Table 4:** Characterization of 1-year post-transplantation outcomes by ECMO status upon multivariate adjustment

**Supplemental Table 5**: 1-year post-transplantation outcomes and complications for all patients by ECMO status and heart failure etiology.

**Supplemental Figure 1**: Stratified log-log survival curves as a function of time to verify proportional hazards assumption.

**Supplemental Table 1.** Demographics and baseline characteristics for all patients by ECMO status and heart failure etiology. Standard deviation for continuous variables and percentage of sample for categorical variables are displayed in parentheses, as appropriate. ECMO = extracorporeal membrane oxygenation, PHM = predicted heart mass

*Denotes statistical significance between ECMO and No ECMO groups within heart failure subgroup based on student t-test, Pearson’s chi-squared test, fisher’s exact, or Wilcoxon rank sum test as appropriate

**Supplemental Table 2.** Comparison of baseline characteristics within ECMO patients based on whether hemodynamic and laboratory data was intact. Patients were defined to have intact data if all seven hemodynamic and laboratory parameters were available in the UNOS registry; if any variable was not available, patient was included in the “Missing Data” group. Standard deviation or percentage of sample are displayed in parentheses, as appropriate. ECMO = extracorporeal membrane oxygenation.

**Supplemental Table 3.** Laboratory and hemodynamic data at the time of transplantation for all patients by ECMO status and heart failure etiology. eGFR was calculated using serum creatinine at the time of transplant according to the Chronic Kidney Disease Epidemiology Collaboration (CKD-EPI) equation; eGFR is presented for all patients including those with preoperative dialysis and then for all patients without preoperative dialysis. Data presented includes only patients with intact hemodynamic data (i.e. not missing any one of seven variables collected). Standard deviation is displayed in parentheses. ECMO = extracorporeal membrane oxygenation, eGFR = estimated glomerular filtration rate

*Denotes statistical significance between ECMO and No ECMO groups within heart failure subgroup based on student t-test or Wilcoxon rank sum test as appropriate

**Supplemental Table 4:** Characterization of 1-year post-transplantation outcomes by ECMO status upon multivariate adjustment. Upon logistic regression, odds ratio for ECMO are presented for each post-transplant outcome after adjustment for age, BMI, months on waitlist, preoperative dialysis, diabetes, prior cardiac surgery, cigarette use, donor age, donor sex, ischemic time, and predicted heart mass. ECMO = extracorporeal membrane oxygenation, CI = confidence interval, BMI = body mass index

**Supplemental Table 5.** 1-year post-transplantation outcomes and complications for all patients by ECMO status and heart failure etiology. Percentage of sample is displayed in parentheses. ECMO = extracorporeal membrane oxygenation

*Denotes statistical significance between ECMO and No ECMO groups within heart failure subgroup based on student t-test or Wilcoxon rank sum test as appropriate

**Supplemental Figure 1**: Stratified log-log survival curves as a function of time to verify proportional hazards assumption. Survival graphs stratified by ECMO status are presented before (left) and after (right) adjustment for covariates, including age, BMI, months on waitlist, preoperative dialysis, diabetes, prior cardiac surgery, cigarette use, donor age, donor sex, ischemic time, and predicted heart mass. Both graphs demonstrate curves that are parallel between the No ECMO and ECMO groups, indicating that the proportional hazards assumption was not violated.
